# CRAC Channel Controls the Differentiation of Pathogenic B Cells in Lupus Nephritis

**DOI:** 10.3389/fimmu.2021.779560

**Published:** 2021-10-22

**Authors:** Xue Li, Qin Zeng, Shuyi Wang, Mengyuan Li, Xionghui Chen, Yuefang Huang, Binfeng Chen, Mianjing Zhou, Yimei Lai, Chaohuan Guo, Siyuan Zhao, Hui Zhang, Niansheng Yang

**Affiliations:** ^1^ Department of Rheumatology, First Affiliated Hospital, Sun Yat-sen University, Guangzhou, China; ^2^ Department of Nephrology, First Affiliated Hospital, Sun Yat-sen University, Guangzhou, China; ^3^ Department of Pediatrics, First Affiliated Hospital, Sun Yat-sen University, Guangzhou, China; ^4^ Institute of Precision Medicine, First Affiliated Hospital, Sun Yat-sen University, Guangzhou, China

**Keywords:** lupus nephritis, store-operated Ca^2+^ entry, Ca^2+^ release-activated Ca^2+^ channel, CaMK2, B-cell differentiation

## Abstract

Store-operated Ca^2+^ release-activated Ca^2+^ (CRAC) channel is the main Ca^2+^ influx pathway in lymphocytes and is essential for immune response. Lupus nephritis (LN) is an autoimmune disease characterized by the production of autoantibodies due to widespread loss of immune tolerance. In this study, RNA-seq analysis revealed that calcium transmembrane transport and calcium channel activity were enhanced in naive B cells from patients with LN. The increased expression of ORAI1, ORAI2, and STIM2 in naive B cells from patients with LN was confirmed by flow cytometry and Western blot, implying a role of CRAC channel in B-cell dysregulation in LN. For *in vitro* study, CRAC channel inhibition by YM-58483 or downregulation by ORAI1-specific small-interfering RNA (siRNA) decreased the phosphorylation of Ca^2+^/calmodulin-dependent protein kinase2 (CaMK2) and suppressed Blimp-1 expression in primary human B cells, resulting in decreased B-cell differentiation and immunoglobulin G (IgG) production. B cells treated with CaMK2-specific siRNA showed defects in plasma cell differentiation and IgG production. For *in vivo* study, YM-58483 not only ameliorated the progression of LN but also prevented the development of LN. MRL/*lpr* lupus mice treated with YM-58483 showed lower percentage of plasma cells in the spleen and reduced concentration of anti-double-stranded DNA antibodies in the sera significantly. Importantly, mice treated with YM-58483 showed decreased immune deposition in the glomeruli and alleviated kidney damage, which was further confirmed in NZM2328 lupus mice. Collectively, CRAC channel controlled the differentiation of pathogenic B cells and promoted the progression of LN. This study provides insights into the pathogenic mechanisms of LN and that CRAC channel could serve as a potential therapeutic target for LN.

## Introduction

Systemic lupus erythematous (SLE) is a prototype of autoimmune disease, which is characterized by the production of a spectrum of autoantibodies including antinuclear antibodies and anti-double-stranded DNA antibodies (1, 2). Lupus nephritis (LN) is one of the most serious complications of SLE. Autoreactive B cells and production of autoantibodies are critical for the initiation and progression of LN (3, 4). Although the therapeutic regimen of LN has been advanced greatly over the past decades, progress on new therapies for LN has been hampered for the poor understanding of disease pathogenesis.

B cells and plasma cells are the source of autoantibodies in autoimmune diseases (5). Memory B cells have been shown to be increased in the blood of SLE patients (6). CD138^+^ plasma cells are also found to be increased in patients with active SLE (7). The activation of autoreactive B cells leads to increased production of autoantibodies, which results in the deposition of immune complexes in the glomeruli, causing kidney damages by the subsequent activation of complement system (8). For the critical role of B cells in the initiation and development of LN, B-cell-targeting therapies should have an important place in the therapeutic management of LN.

Calcium signals control a vast array of cellular activities and physiological functions. Mutations in Bruton’s tyrosine kinase (BTK) leads to defects in inositol 1,4,5-trisphosphate (IP3)-mediated calcium signaling, resulting in low numbers of mature B cells and a lack of immunoglobulin production (9). Loss of IP3-receptor-mediated Ca^2+^ release in mouse B cells results in abnormal B-cell development and function (10). Mice with a gain-of-function mutation in PLCγ2, a Ca^2+^-dependent lipase that hydrolyzes phosphatidylinositol 4, 5-bisphosphate (PIP2) to produce IP3, are shown to develop multiorgan autoimmune inflammatory disease with increased Ca^2+^ responses in B cells (11). Dysregulated calcium signaling is involved with the pathophysiological processes in several autoimmune and inflammatory diseases (12) and in SLE (13). It has been shown that B-cell receptor (BCR)-mediated early signal transduction events in B cells from SLE patients are abnormal and lead to increased calcium signals in these B cells (14), pinpointing the important role of calcium signaling in B cell activation in LN.

Store-operated Ca^2+^ entry (SOCE) is the main Ca^2+^ influx pathway in lymphocytes and is essential for immune response (12, 15, 16). SOCE is mediated by Ca^2+^ release-activated Ca^2+^ (CRAC) channels, which are comprised of stromal interaction molecule (STIM) and calcium release-activated calcium modulators (ORAI) (17, 18). Mutations in genes encoding the CRAC channel abolish SOCE in cells of the immune system and cause severe combined immunodeficiency (17, 19). Calcium signaling *via* ORAI1 has been involved in the pathogenesis of autoimmune diseases by driving Th17 differentiation (20). *STIM1* deficiency significantly reduced Th1/Th17 responses and resulted in complete protection from experimental autoimmune encephalomyelitis (21). Compared to T cells, the roles of CRAC channel in B cells are far less clear. How CRAC channel affects B cells and its pathogenic roles in LN is not clear.

Ca²⁺/calmodulin (CaM)-dependent protein kinase2 (CaMK2) is a serine-/threonine-specific protein kinase that is regulated by the Ca²⁺/CaM complex (22). CaMK2 has been involved in many signaling pathways and is necessary for Ca²⁺ homeostasis, T-cell development, and activation (23, 24). CaMK4, another member of the CaMK family, has been shown to compromise podocyte function and promote renal diseases in LN (25, 26). Inhibition of CaMK4 decreases the frequency of Th17 cell differentiation and ameliorates lupus-like disease in murine model (27). Previous data reveal that CaMK2 promotes proteinuria by regulating kidney podocytes (28). However, the roles of CaMK2 in human B cells and its contribution to LN is not clear yet.

In the current study, we found that CRAC-channel-mediated calcium signaling is enhanced in B cells from patients with LN. CRAC channel inhibition by YM-58483 or knockdown of CRAC channel by ORAI1- and STIM2-specific siRNA led to suppression of CaMK2 signaling and decreased B-cell differentiation. Lupus mice treated with YM-58483 showed reduced anti-double-stranded DNA antibodies (anti-dsDNA), decreased immune deposition in the glomeruli, and improved renal function. Taken together, CRAC channel mediates the development and progression of LN by promoting the differentiation of B cells into plasma cells.

## Methods and Materials

### Patient Samples

Patients with SLE were recruited from the First Affiliated Hospital, Sun Yat-sen University, who fulfilled the American College of Rheumatology criteria for the classification of SLE with renal damages confirmed by renal biopsy or defined as proteinuria >0.5 g/24 h or ≥3+ (29). Patients with rheumatoid arthritis (RA) (30) or primary Sjögren’s syndrome (pSS) (31) were recruited as disease controls. Patients with preexisting cancer, pregnancy, or acute and chronic infections were excluded from this study. The demographic data are summarized in **Supplementary Table S1**. Age- and sex-matched blood samples were obtained from Guangzhou Blood Center from healthy donors. Clinical disease activity was scored using SLE Disease Activity Index (SLEDAI) scoring system (32). Informed consents were obtained from all patients. The study was conducted in accordance with recognized ethical guidelines of Declaration of Helsinki.

### Cell Isolation

Peripheral blood mononuclear cells (PBMCs) were isolated from blood samples of patients with LN or healthy controls (HC) by density gradient centrifugation. CD19^+^ B cells were purified from PBMCs by positive selection using the EasySep™ Human CD19 Positive Selection Kit. Naive B cells were isolated using EasySep™ Human Naive B Cell Isolation Kit (STEMCELL Technologies). Cell purities were checked by FACS (>95%).

### B-Cell Transfections

Isolated human B cells were transfected with ORAI1- or CaMK2-specific small-interfering RNA (siRNA) (RiboBio) using a NucleofectorTM Kit (Lonza) according to the manufacturer’s protocols as described (33). Cells were then let to rest overnight to recover from the electroporation. Knockdown efficiency was confirmed by Western blot.

### B-Cell Culture

For B-cell differentiation, isolated naive B cells were cultured in Roswell Park Memorial Institute (RPMI) 1640 supplemented with 10% fetal bovine serum (FBS) (Life Technologies) and 1% penicillin/streptomycin (Life Technologies). Cells were stimulated with antihuman IgM (5 μg/ml, Sigma, #10759) and antihuman CD40 (1 μg/ml, Bioxcell, #BE0189) antibodies in the presence of interleukin (IL)-2 (20 ng/ml, PeproTech), IL-4 (25 ng/ml, Sino Biological) and IL-21 (50 ng/ml, Sino Biological) for 8 days.

For signaling study, B cells were stimulated with antihuman IgM (5 μg/ml) and antihuman CD40 (1 μg/ml) antibodies for 72 h. CRAC channel inhibitor YM-58483 (50 nM, MedChemExpress, USA) or CaMK2 inhibitor KN-93 (5 μM, Selleck) was included in some of the experiments. Cells were maintained in a humidified atmosphere at 37°C with 5% CO_2_. Cells were collected for Western blot or FACS analysis.

### Ca^2+^ Measurement

To measure basil level of Ca^2+^ in B cells, cells were incubated with Fluo-3, AM (Sigma) in Ca^2+^-free buffer at 37°C for 30 min and detected by flow cytometry. To detect Ca^2+^ influx in B cells, cells were first labeled with Fluo-3, AM and run by flow cytometry in buffer with Ca^2+^. Antihuman IgM antibody (10 μg/ml) was added to stimulate Ca^2+^ influx during measurement. YM-58483 was used to block the influx of Ca^2+^ in some of the experiments.

### Western Blot

B cells were processed for Western blot analysis as we described previously (34). Proteins were loaded to sodium dodecyl sulfate (SDS)-polyacrylamide gels and electrotransferred onto polyvinylidine difluoride membranes after separation. Membranes were blocked with 5% bovine serum albumin in TBST buffer and incubated with primary antibodies against ORAI1 (Abcam, #ab59330), ORAI2 (Abcam, #ab180146), STIM2 (ABclonal, #A17743), Blimp-1 (BD Bioscience, #564703), and p-CaMK2 (Abcam, #ab171095), at 4°C overnight. Membranes were incubated with anti-GAPDH primary antibody (Cell Signaling Technology, #2118s) and used as internal control. Membranes were then washed with TBST and incubated with horseradish-peroxidase-conjugated antirabbit (Cell Signaling Technology, #7074S) or antimouse IgG (Cell Signaling Technology, #7076S) at room temperature for 60 min. Signals were detected by enhanced chemiluminescence analysis kit.

### Flow Cytometry

For human B cell staining, cells were stained with PE-Cy7-conjugated anti-CD19 (BioLegend, #302216), PE-conjugated CD27 (BioLegend, #302808), BV421-conjugated IgD (BioLegend, #348226), BV421-conjugated anti-CD138 (BioLegend, #356516), and APC-conjugated anti-IgG antibodies (BioLegend, #304136). Alternately, cells were permeabilized and stained with primary antibodies against ORAI1, ORAI2, and STIM2 at 37°C for 30 min. Cells were then washed with phosphate-buffered saline (PBS) and incubated with Alexa Fluor^®^ 488 Goat antirabbit IgG (Thermo Fisher, #A-11008) secondary antibodies at room temperature for 30 min. For mouse cell staining, cells were stained with APC-conjugated B220 (BioLegend, #318326), PE-Cy7-conjugated CD38 (BioLegend, #102718), BV421-conjugated CD138 (BioLegend, #142523), PE-conjugated PD-1 (BioLegend, #135205), APC-conjugated CXCR5 (BioLegend, #145506), PE-conjugated CD95 (BioLegend, #152608), fluorescein isothiocyanate (FITC)-conjugated GL-7 (BioLegend, #144612), APC-Cy7-conjugated-CD4 (BioLegend, #100526), BV510-conjugated-CD3 (BioLegend, #100234), Percp-cy5.5-conjugated-CD45 (BioLegend, #103132), APC-conjugated-CD8 (BioLegend, #100712), PE-Cy7-conjugated-CD19 (BioLegend, #506921) and BV605-conjugated-CD11b (BioLegend, #101257). Samples were analyzed using a BD LSR Fortessa (BD Bioscience).

### B-Cell Proliferation Assay

Isolated human naive B cells were stained with 5 μM of carboxyfluorescein succinimidyl ester (CFSE, Thermo Fisher) at 37°C for 10 min. Cells were then incubated with precold PBS on ice for 5 min to stop the reaction. Cells were then washed twice and seeded to 96-well plates in the presence of antihuman IgM (5 μg/ml) and antihuman CD40 (1 μg/ml) antibodies and IL-2 (20 ng/ml) for 4 days. Proliferation was measured by flow cytometry according to the dilution of CFSE as we described before (35).

### qPCR

Trizol (Invitrogen) was used to isolate RNA from human B cells. RNA was then reverse transcribed into complementary DNA (cDNA) using a reverse transcript kit (Accurate Biotechnology). cDNA was amplified in a SYBR green-based quantitative RT-PCR using SYBR Green qPCR Kit (Accurate Biotechnology). Primers for human *CaMK1*, *CaMK2d*, *CaMK2g*, and *CaMK4*, and *GAPDH* were used: *CaMK1*, 5′- GGATTGCTGGTCCATAGGTGTC-3′ (forward), 5′-CAGAGATGTCGTCCCAGTAAGG-3′ (reverse); *CaMK2d*, 5′- ACACGGTGACTCCTGAAGCCAA-3′ (forward), 5′- GTCTCCTGTCTGTGCATCATGG-3′ (reverse); *CaMK2g*, 5′- GACACGGTAACTCCTGAAGCCA3′ (forward), 5′-TCCACAGTCTCCTGACGATGCA-3′ (reverse); *CaMK4*, 5′-GTTCTTCTTCGCCTCTCACATCC-3′ (forward), 5′- CTGTGACGAGTTCTAGGACCAG-3′ (reverse); and *GAPDH*, 5′-GGAGCGAGATCCCTCCAAAAT-3′ (forward), 5′- GGCTGTTGTCATACTTCTCATGG-3′ (reverse). For amplification, cDNA was denatured at 95°C for 30 s and amplified for 40 cycles at 95°C for 5 s and 60°C for 30 s. Transcripts for individual genes were normalized to GAPDH transcripts and presented as relative expression.

### Proteinuria, Blood Urea Nitrogen, and Creatinine

Proteinuria were detected with proteinuria analysis strips (Multistix 10SG). The severity of proteinuria was classified into six levels as: negative, 10, 30, 100, 300, and ≥2000 mg/dl protein. Mouse urine samples with protein ≥300 mg/dl was defined as severe proteinuria. Blood urea nitrogen (BUN) and creatinine concentrations in mouse sera were determined by using a commercial autoanalyzer (Beckman Coulter).

### Transmission Electron Microscopy

Mice were euthanized using a carbon dioxide chamber. Mouse kidneys were then perfused with prewarmed normal saline. Kidney samples were fixed in 2.5% glutaraldehyde and 2% paraformaldehyde (wt/vol) in 0.1M phosphate buffer pH 7.4, processed for electron microscopy and examined with a Tecnai G2 Spirt Twin electron microscope as we described before (36).

### Histopathology

Mouse kidneys were fixed in 10% neutral formalin, dehydrated, and embedded with paraffin. Tissue blocks were sectioned (2 µm) and followed by periodic acid–Schiff (PAS) staining. Histopathological evaluation was performed on a scale of 0–3 according to cell proliferation and cell infiltration by two observers blind to the protocol as previously described (37).

To assess immune deposition in the glomeruli, kidney samples were collected and embedded in optimal cutting temperature (OCT) compound (SAKURA). Renal blocks were sectioned (4 μm), and slides were fixed in pre-cold acetone for 10 min. Tissue slides were then washed with PBS twice and incubated with FITC-conjugated antihuman IgG (Thermo Fisher, #A11001) and FITC-conjugated antihuman complement component 3 (C3) antibodies (Thermo Fisher, #PA1-28933) at 4°C overnight. Sections were washed and counterstained with 4′,6-diamidino-2-phenylindole (DAPI) in mounting medium (Vector Laboratories). Fluorescence signals were examined using a fluorescence microscopy (Olympus).

### ELISA

Serum anti-DNA level was determined by anti-dsDNA enzyme-linked immunosorbent assay (ELISA) Kit (AMEKO) according to the manufacturer’s protocol.

### Mouse Experiments

Female MRL/*lpr* mice were obtained from SLAC Laboratory Animal Company. NZM2328 mice were a gift from Dr. SM Fu from University of Virginia. All of the mice were maintained under specific pathogen-free condition at the Experimental Animal Center of Sun Yat-sen University. Mice were randomized divided into vehicle- or YM-58483-treated group. Mice were treated with YM-58483 (1 mg/kg) or equal volume of vehicle intraperitoneal daily for 6 weeks. Mice were monitored for the potential therapeutic-related toxicities, and no signs of toxicities related to YM-58483 treatment was recorded (**Supplementary Figure S1**). For disease treatment, MRL/*lpr* mice were treated from 12 weeks of age after the development of proteinuria. For disease prevention, MRL/*lpr* mice were treated from 8 weeks of age before the development of proteinuria. At the end of treatment, mice were euthanized. Spleen and kidney samples were collected for single cell analysis or pathological evaluation. Mouse spleens were collected, cut into 3–5 mm pieces, and smashed using a syringe plunger to prepare single cell suspension. ACK lysing buffer was used to remove red blood cells from splenocytes. Cells were then filtered through a 70-µm nylon cell strainer (BD Pharmingen).

### RNA-Seq and Bioinformatics

Naive human B cells were isolated from three donors and stimulated with antihuman IgM (5 μg/ml) and antihuman CD40 (1 μg/ml) antibodies in the presence of YM-58483 or vehicle for 72 h. RNA purification was performed using RNeasy Kit (Qiagen), and DNA was removed by DNase digestion. RNA quality was defined by RIN and OD260/280. The RNA-seq libraries were made with the Ovation^®^ Ultralow Library Systems and sequenced on an Illumina HiSeq at POHC@IGB. Human reference genome (hg38) was used for alignment using STAR. Aligned reads were mapped to known genes. The samples were compared in terms of their total reads mapped to genes, distribution of reads across genes, and global correlation with each other.

Fifty picograms of total RNA was used as input for the SMART-seq v3 cDNA synthesis kit (Takara) using 10 cycles of PCR amplification. One nanogram of cDNA was used as input for the NexteraXT kit (Illumina) using nine cycles of PCR amplification. Final libraries were quantitated by qPCR and sized distribution determined by a bioanalyzer prior to pooling and sequencing on a HiSeq2500 using 50 bp PE chemistry. RAN-seq data generated in this study is available at GEO data base (GSE175424).

RNA-seq data of B cell purified from peripheral blood of patients with SLE or HC (GSE118254) were acquired from NCBI GEO database (https://www.ncbi.nlm.nih.gov/geo/) (38). The dataset was based on GPL16791 (Illumina HiSeq 2500, *Homo sapiens*), containing 83 samples (including 7 SLE and 6 HC active naive B cells samples, 8 SLE and 7 HC switched memory B cells).

“fastqc (version 0.11.9)” for the quality assessment of raw RNA-seq reads, “hisat2 (version 7.5.0)” for the sequencing reads alignment to the reference human genome (version hg38, UCSC Genome Browser), and “featureCounts (version 2.0.1)” for transcripts assemble and expression levels calculation were adopted.

### Identification of DEGs

For RNA-seq data acquired from GEO database or data from YM-58483 or vehicle-treated samples, Statistical software R (version 4.0.1, https://www.r-project.org/) and Bioconductor packages (available online: http://www.bioconductor.org/) were applied to screen differential expression genes (DEGs). “DESeq2” package was used for DEG analysis, and *p* < 0.1 and |log2foldchange| > 0 were the cutoff criteria for the identification of DEGs. For RNA-seq data acquired from GEO database, the top 200 DEGs ordering by |log2foldchange| were chosen to picture a heat map.

### Gene Ontology and Pathway Enrichment Analysis

For RNA-seq data acquired from GEO database, DAVID 6.8 (available online: https://david.ncifcrf.bov/) was applied to analyze the molecular function (MF), cellular component (CC), biological process (BP), and Kyoto Encyclopedia of Genes and Genomes (KEGG) of the top 200 DEGs. A value of *p* < 0.1 was considered statistically significant. A bubble plot of top 20 enriched GO and KEGG terms was drawn by using “ggplot2” R package. Focusing on terms related to calcium transport, we screen DEGs in these terms to picture a heat map and violin plots of interested genes. p < 0.1 was considered reaching statistic significant level.

### Gene Set Enrichment Analysis

For RNA-seq data acquired from GEO database or data from YM-58483 or vehicle-treated samples, Gene Set Enrichment Analysis (GSEA) enrichment scores (ES) using interested gene sets (“GO_CALCIUM_ION_TRANSMEMBRANE_TRANSPORT” and “KEGG_CALCIUM_SIGNALING_PATHWAY”) was calculated for seven SLE and six HC phenotype. For the three YM-58483 and three vehicle phenotype, ES was calculated using interested gene sets (“GO_CALCIUM_ION_TRANSMEMBRANE_TRANSPORT”, “KEGG_CALCIUM_SIGNALING_PATHWAY,” “PID_NFAT_3PATHWAY,” “GO_NIK_NF_KAPPAB_SIGNALING,” and “GO_TOR_SIGNALING” that are available at molecular signature database (https://www.gsea-msigdb.org/gsea/msigdb/index.jsp), and “GO: 0042100” available at GENEONTOLOGY (http://geneontology.org/). *p* < 0.1 was considered reaching statistic significant level.

### Statistical Analysis

Data are presented as means ± standard error of mean (SEM). Statistical analysis was performed using Prism 5.0. Comparisons were assessed using either the Student’s t-test, paired Student’s t-test or one-way ANOVA with or without repeated measurements followed by Bonferroni’s multiple comparison post-test, as appropriate. Unless otherwise stated, *p* < 0.05 were considered as statistically significant.

## Results

### B Cell Dysregulation and Bioinformatic Analysis of RNA-Seq Data From SLE Patients

**Figure 1:**
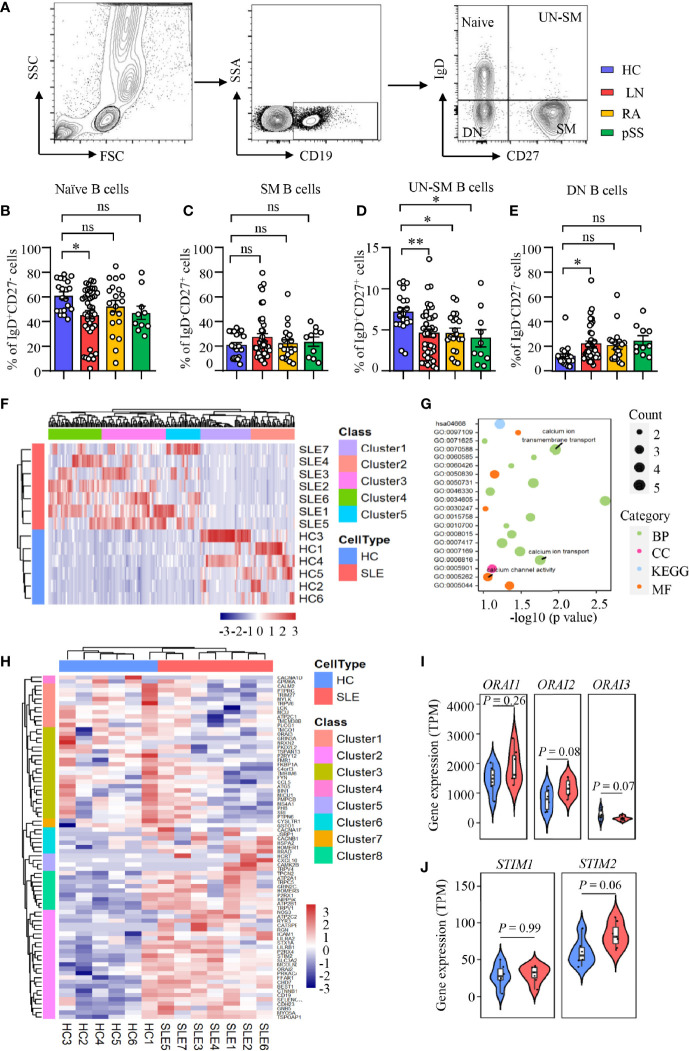
Enriched calcium signaling in B cells from patients with LN. **(A–E)** PBMCs were isolated from patients with lupus nephritis (LN), rheumatoid arthritis (RA), primary Sjogren’s syndrome (pSS), or healthy controls (HC). Naive B cells (CD19^+^IgD^+^CD27^−^), switched memory B cells (CD19^+^IgD^−^CD27^+^, SM), unswitched memory B cells (CD19^+^IgD^+^CD27^+^, UN-SM), and double-negative B cells (CD19^+^IgD^−^CD27^−^, DN) were analyzed by flow cytometry. **(A)** Gating strategy. Percentages of **(B)** naive B cells, **(C)** SM B cells, **(D)** UNSM B cells, and **(E)** DN B cells are summarized in dot plot with bar plot. LN = 40, RA = 20, pSS = 10, HC = 20. **(F–J)** RNA-seq data of B cell purified from peripheral blood of patients with systemic lupus erythematosus (SLE) or HC were acquired from the database (GSE118254) for gene expression and bioinformatics analysis. **(F)** Gene expression clustered heatmap represents the scaled average expression of top 200 DEGs. **(G)** Bubble plot of GO and KEGG analysis. The number of DEGs enriched in different terms are indicated by size of dots. **(H)** Clustered heatmap of the scaled average expression 79 DEGs in three calcium-related GO classes. **(I, J)** Violin plot of ORAI1, ORAI2, ORAI3, STIM1, and STIM2 expression levels. The upper, median, and lower horizontal lines of white box represent 75th, median, and 25th percentage of the gene expression distribution, respectively. All data are mean ± SEM. *p < 0.05 and **p < 0.01 by one-way ANOVA in Panel **(B–E)** and Student’s t-test in Panels **(I, J)** ns, not significant.

LN is characterized by the production of autoantibodies by B cells or plasma cells. B-cell-targeted therapy has been effective in the treatment of LN (39). B cells from human peripheral blood can be categorized into four distinct fractions based on the expression of IgD and CD27: IgD^+^CD27^−^ naive B cells, IgD^−^CD27^+^ switched memory (SM) B cells, IgD^+^CD27^+^ unswitched memory (UN-SM) B cells, and IgD^−^CD27^−^ double-negative (DN) B cells (**Figure 1A**). We found that naive population was decreased in patients with LN, while the naive B cells were not different in RA and pSS patients (**Figure 1B**). SM B cells were not different between patients with LN, RA, pSS, and HC (**Figure 1C**). Interestingly, UN-SM B cells were found to be decreased in patients with LN, RA, and pSS (**Figure 1D**). DN B cells were only observed to be increased in patients with LN (**Figure 1E**).

To investigate the underlying signaling that drive B-cell dysregulation in LN, RNA-seq analysis was performed as we described in *Methods and Materials* to study the differential gene expressions and enriched signaling in SLE B cells. Comparing to HC, we found 2,757 upregulated and 2,059 downregulated differentially expressed genes (DEGs) in active naive B cells (CD19^+^IgD^+^CD27^−^MTG^+^CD24^−^CD38^−^) from SLE patients (p < 0.1). The top 200 DEGs in naive B cells from patients with SLE or HC were shown as heat map ordered by absolute value of log2 (fold change). The result demonstrated that samples from the two groups could be divided into two different categories, indicating great difference in gene expression in naive B cells between patients with SLE and HC (**Figure 1F**). We put the 200 DEGs into GO and KEGG analysis and clustered 35 classes, including 20 biological processes (BF), 7 cellular components (CC), 6 molecular function (MF), and 2 KEGG pathway (p < 0.1).

After ordered by fold enrichment, the top 20 classes were displayed in a bubble plot. The result showed that three GO classes were related to calcium ion transport (GO: 0070588 calcium ion transmembrane transport, GO: 0006816 calcium ion transport, GO: 0005262 calcium channel activity) (**Figure 1G**). We focused on the calcium-related classes and performed a further analysis. The heat map showed a clustering of 79 DEGs in the three GO terms we focused (GO: 0005262, GO: 0006816, and GO: 0070588), which demonstrated that the two groups of samples could be divided into two different categories, and DEGs could be clustered into eight categories. Of these 79 DEGs, 44 genes were upregulated in naive B cells from SLE patients [log2 (fold change) > 0, p <0.1] (**Figure 1H**). Furthermore, we found that CRAC channel associated genes of *ORAI2* and *STIM2* were increased significantly at p < 0.1 level in naive B cells from SLE patients. *ORAI3* was downregulated in naive B cells from SLE patients. The expression level of *ORAI3* was very low in all samples (**Figures 1I, J**). However, genes were not different in CD4 T cells during calcium transmembrane transport (“GOMP_CALCIUM_ION_TRANSMEMBRANE_TRANSPORT”), calcium ion transport (“GOMP_CALCIUM_ION_TRANSPORT”), and calcium channel activity (“GOMF_CALCIUM_CHANNEL_REGULATORY_ACTIVITY”) in SLE phenotype by GESA analysis (**Supplementary Figure S2**).

### Increased CRAC Channel Components in B Cells From LN Patients

**Figure 2:**
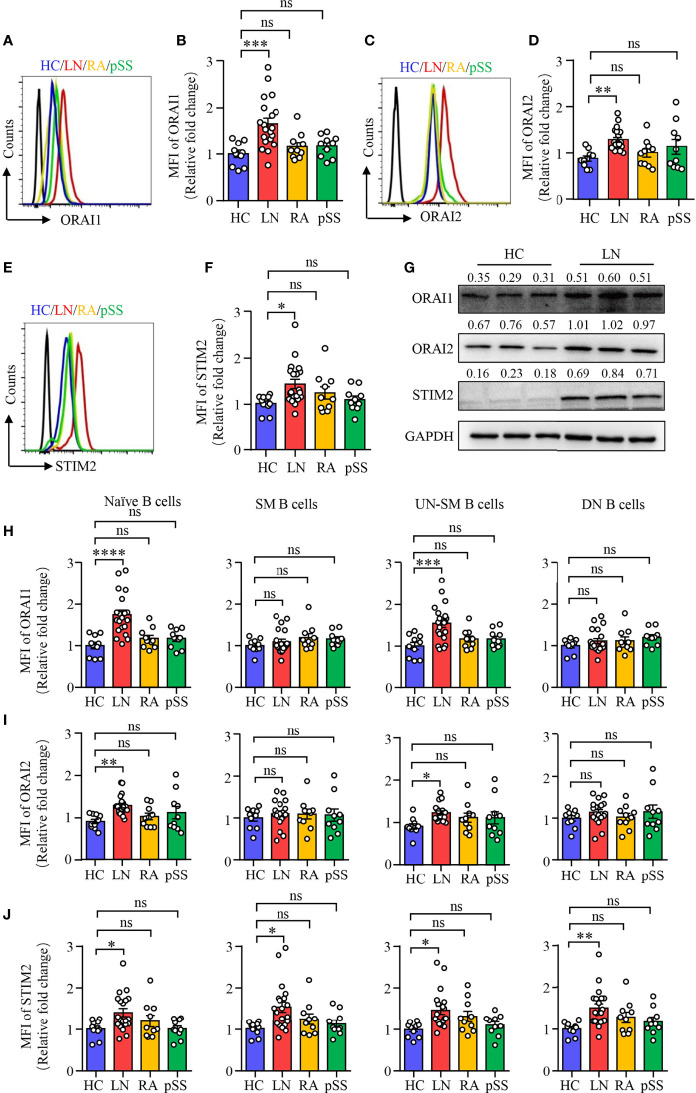
Increased CRAC channel in B cells from patients with LN. PBMCs were isolated from patients with lupus nephritis (LN), rheumatoid arthritis (RA), primary Sjogren’s syndrome (pSS), or healthy controls (HC). Orai1, Orai2, and STIM2 expression in B cells was measured by flow cytometry or Western blot. **(A–F)** ORAI1, ORAI2, and STIM2 expression in B cells from LN, RA, pSS, or HC was measured by flow cytometry. Representative histograms were gated on CD19^+^ B cells. Mean fluorescence intensity (MFI) of ORAI1, ORAI2, and STIM2 from LN (n = 20), RA (n = 10), pSS (n = 10), or HC (n = 10) were summarized. **(G)** CD19^+^ B cells were isolated from LN or HC. ORAI1, ORAI2, and STIM2 expression in B cells were measured by Western blot. GAPDH was used as internal control, and relative expression values are indicated above each lane. Representative bands are shown. **(H–J)** Mean fluorescence intensity (MFI) of Orai1, Orai2, and STIM2 in naive B cells (CD19^+^IgD^+^CD27^−^), switched memory B cells (CD19^+^IgD^−^CD27^+^, SM), unswitched memory B cells (CD19^+^IgD^+^CD27^+^, UNSM), and double-negative B cells (CD19^+^IgD^−^CD27^−^, DN) were analyzed by flow cytometry and are summarized in dot plot with bar plot. All data are mean ± SEM. *p < 0.05, **p < 0.01, ***p < 0.001 and ****p < 0.0001 by one-way ANOVA. ns, not significant.

To further confirm the RNA-seq data, B cells were isolated from patients with LN or HC. The expression of ORAI1, ORAI2, and STIM2 was measured by flow cytometry. In consistent with the RNA-seq data, the expression of ORAI1, ORAI2, and STIM2 was increased significantly in B cells from patients with LN at the protein level. The expression of ORAI1, ORAI2, and STIM2 in B cells from patients with RA or pSS was not different compared to that of HC (**Figures 2A–F**). The expression of ORAI1, ORAI2, and STIM2 in B cells from LN or HC was further measured by Western blot. Similar results were found that further confirmed the increased expression of CARC channel components in B cells from LN (**Figure 2G**). The increased CRAC channel in B cells from patients with LN was followed by increased Ca^2+^ level (**Supplementary Figures S3A, B**). Ca^2+^ influx by BCR stimulation was increased in B cells from LN patients (**Supplementary Figure S3C**). Interestingly, the expression of ORAI1, ORAI2, and STIM2 in B cells from LN patients was correlated with disease activities as determined by SLEDAI (**Supplementary Figures S3D–F**), suggesting the important role of CRAC channel in the pathogenesis of LN by regulating B-cell activation.

To further explore and characterize the expression of CRAC channel components in B-cell subsets described in **Figure 1**, we measured ORAI1, ORAI2, and STIM2 expression in the B cell subsets by flow cytometry. The expression of all these three CRAC channel components was increased in naive B cells and UN-SM B cells from patients with LN. However, other than STIM2 in SM and DN B cells, the expression of all these three CRAC channel components in SM and DN B cells were largely unchanged in patients with LN when compared to that of HC (**Figures 2H–J**). Although the percentage of UN-SM B cells was decreased in patients with RA and pSS, we did not find the changed expression of ORAI1, ORAI2, and STIM2 in these B-cell fractions compared to that of HC (**Figures 2H–J**), indicating that B cells might be regulated by different signals in RA and pSS.

### CRAC Channel Inhibition Suppresses B-Cell Differentiation

**Figure 3:**
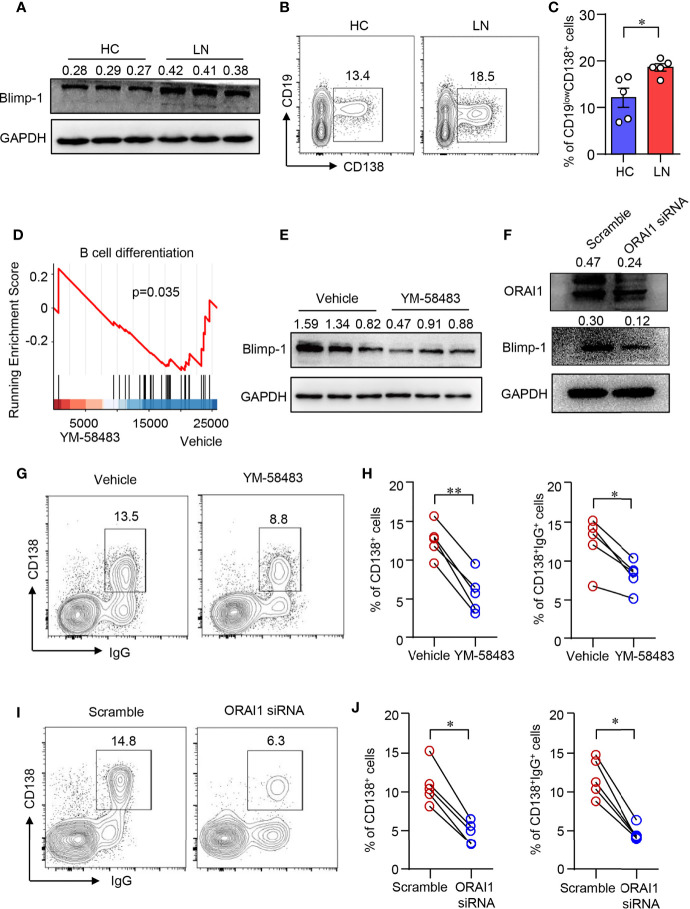
CRAC channel inhibition suppressed B cell differentiation. **(A)** Blimp-1 expression in B cells from patients with lupus nephritis (LN) or healthy controls (HC) was measured by Western blot and representative bands of six independent samples. **(B, C)** Naive B cells from LN or HC were cultured in the presence of anti-IgM (5 μg/ml), anti-CD40 (1 μg/ml), IL-2 (20 ng/ml), IL-4 (20 ng/ml), and IL-21 (50 ng/ml) for 8 days. Percentages of CD19^low^CD138^+^ plasma cells were determined by flow cytometry. Representative counter plots are shown and data from five samples. Data are mean ± SEM. *p < 0.05 by t-test. **(D)** GSEA plot for the gene set of B-cell differentiation showing the enrichment scores for YM-58483 or vehicle-treated B cells (n = 3). **(E)** Blimp-1 expression in B cells treated with YM58483 or vehicle was measured by Western blot and representative bands of six independent samples. **(F)** Blimp-1, ORAI1 expression in B cells treated with ORAI1 siRNA or scramble siRNA was measured by Western blot. Representative bands of six samples are shown. **(G, H)** CD138 and IgG expression in B cells treated with YM-58483 or vehicle. Representative counter plots are shown, and the percentages of CD138^+^ or CD138^+^IgG^+^ cells are summarized in dot plot with bar plot (n = 5). **(I, J)** CD138 and IgG expression in B cells treated with ORAI1 siRNA or scramble siRNA. Representative counter plots are shown, and the percentage of CD138^+^ or CD138^+^IgG^+^ cells are summarized in dot plot with bar plot (n = 5). For the Western blot data, relative expression values to GAPDH are indicated above each lane. *p < 0.05, **p < 0.01 by paired t-test.

Blimp-1 is the key transcription factor that drives the terminal differentiation of B cells into plasma cells (40). We found that Blimp-1 expression was profoundly increased in B cells from LN as measured by Western blot (**Figure 3A**). Furthermore, naive B cells from patients with LN were prone to differentiate into CD19^low^CD138^+^ plasma cells as induced *in vitro* (**Figures 3B, C**). To investigate whether CARC channel regulates B-cell differentiation, YM-58483 was used to block CRAC channel in B cells during the induction of B cell differentiation *in vitro*. The inhibition of Ca^2+^ influx into B cells by YM-58483 was confirmed by flow cytometry (**Supplementary Figure S4**). We first performed an RNA-seq to screen the effects of YM-58483 over B-cell activation and differentiation. We found that B-cell differentiation was inhibited by YM-58483 as by RNA-seq analysis (**Figure 3D**), indicating that store-operated CRAC channel might be associated with B-cell differentiation. Blimp-1 expression in B cells was inhibited by YM-58483 significantly as measured by Western blot (**Figure 3E**). To further confirm the regulation of CRAC channel over B cells, B cells were transfected with ORAI1-specific siRNA. Knockdown efficiency was confirmed by Western blot (**Figure 3F**). We found that knockdown of ORAI1 decreased the expression of Blimp-1 as measured by Western blot (**Figure 3F**). As for B-cell differentiation, YM-58483 decreased the differentiation of plasma cells from 12.59% to 5.73%. YM-58483 also decreased IgG production by differentiated plasma cells from 12.37% to 8.17% (**Figures 3G, H**). Knockdown of ORAI1 effectively suppressed B-cell differentiation and IgG production (**Figures 3I, J**). However, B-cell proliferation was not affected by YM-58483 (**Supplementary Figures S5A, B**). These data demonstrated that CRAC channel is important for B-cell differentiation and IgG production.

### CRAC Channel Regulates B-Cell Differentiation Through CaMK2

**Figure 4:**
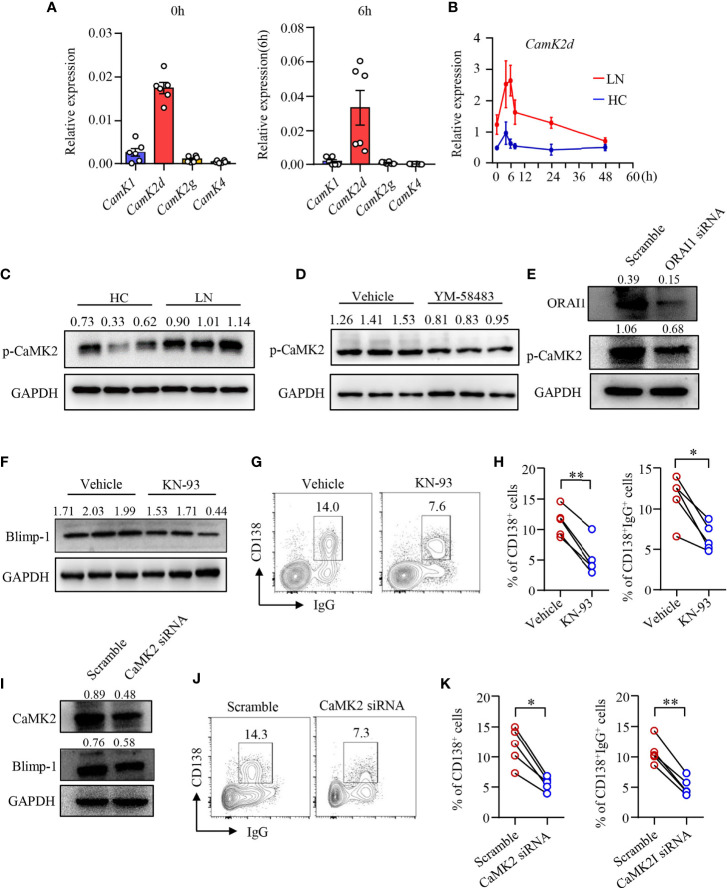
CRAC channel suppressed B-cell differentiation by inhibiting CaMK2. **(A)** qPCR analysis of *CaMK1*, *CaMK2d*, *CaMK2g*, and *CaMK4* mRNA expression in B cells from healthy controls (HC) stimulated by anti-IgM (5 μg/ml) and anti-CD40 (1 μg/ml) at 0 and 6 h time points. **(B)** qPCR analysis of *Camk2d* mRNA expression in B cells from patients with lupus nephritis (LN) or healthy controls (HC). Data were normalized to GAPDH (n = 6). Data are mean ± SEM. **(C)** Phosphorylation of CaMK2 (p-CaMK2) in B cells from patients with LN or HC was measured by Western blot. Representative bands of six samples are shown. **(D)** p-CaMK2 in B cells treated by YM-58483 (20 nM) or vehicle was measured by Western blot. Representative bands of six samples are shown. **(E)** ORAI1 and p-CAMK2 expression in B cells treated with ORAI1- or scramble-siRNA was measured by Western blot. Representative bands of six samples are shown. **(F)** Blimp-1 expression in B cells treated with KN-93 (1 μM) or vehicle was measured by Western blot. Representative bands are shown. **(G, H)** CD138 and IgG expression in B cells treated with YM-58483 or vehicle. Representative counter plots are shown, and the percentages of CD138^+^ or CD138^+^IgG^+^ cells are summarized in dot plot with bar plot (n = 5). **(I)** CaMK2 and Blimp-1 expression in B cells treated with CaMK2- or scramble-siRNA was measured by Western blot. Representative bands of six samples were shown. **(J, K)** CD138 and IgG expression in B cells treated with CaMK2- or scramble-siRNA. Representative counter plots are shown, and the percentage of CD138^+^ or CD138^+^IgG^+^ cells are summarized in dot plot with bar plot (n = 5). For the Western blot data, relative expression values to GAPDH are indicated above each lane. All data are mean ± SEM. *p < 0.05, **p < 0.01 by paired t-test.

Upon increased intracellular Ca^2+^, CaM responses and alters the functionality of numerous proteins including a family of protein kinases CaMKs (41). We found that the expression of *Camk2d* was among the most abundant CaMK gene in human B cells as determined by qPCR (**Figure 4A**). Camk2d, as one of the key CaMKs activated by CaM, was upregulated in B cells from LN patients (**Figure 4B**). Furthermore, the phosphorylation of CaMK2 was significantly increased in naive B cells from patients with LN (**Figure 4C**). To investigate whether CRAC channel regulates CaMK2 in human B cells, human B cells were treated with CRAC channel inhibitor YM-58483. We found that the phosphorylation of CaMK2 was inhibited by YM-58483 significantly (**Figure 4D**). B cells were further treated with ORAI1 siRNA by electroporation. B cells treated with ORAI1 siRNA showed decreased phosphorylation of CaMK2 significantly as measured by Western blot (**Figure 4E**).

To further study the roles of CaMK2 in human B-cell differentiation, human B cells were first treated with CaMK2 inhibitor KN-93 during the induction of plasma cell differentiation. We found that KN-93 inhibited Blimp-1 expression in B cells as measured by Western blot (**Figure 4F**). B-cell differentiation and IgG production by differentiated plasma cells were also reduced by KN-93 (**Figures 4G, H**). To further confirm the role of CaMK2 in B-cell differentiation, CaMK2 expression was knocked down by CaMK2-specific siRNA (**Figure 4I**). The results revealed that knockdown of CaMK2 not only decreased Blimp-1 expression but also suppressed the differentiation of B cells into plasma cells as measured by Western blot and flow cytometry, respectively (**Figures 4I–K**). IgG production was also reduced by the knockdown of CaMK2 significantly as measured by flow cytometry (**Figures 4J, K**). However, the proliferation of B cells was not affected by KN-93 (**Supplementary Figures 5C, D**).

### YM-58483 Decreases B-Cell Differentiation and Immune Complex Deposition in the Kidney of MRL/*lpr* Mice

**Figure 5:**
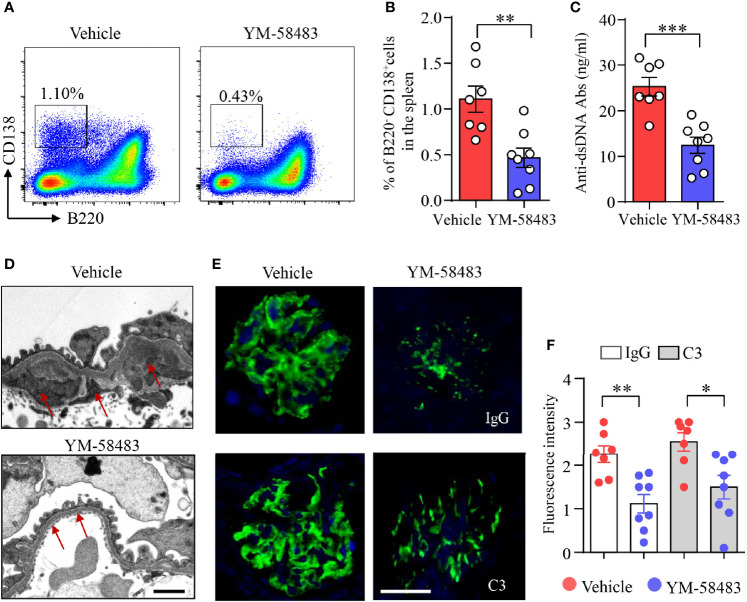
YM-58483 reduced immune deposition in the glomeruli by suppressing B-cell differentiation in MRL/lpr lupus mice. MRL/lpr mice (12-week) were treated with YM-58483 (1 mg/kg) or vehicle intraperitoneal once a day for 6 weeks as described in *Materials and Methods*. **(A, B)** B220^-^CD138^+^ plasma cells in the spleen of YM-58483- or vehicle-treated mice was determined by flow cytometry. Representative pseudocolor plots are shown. The percentages of B220^-^CD138^+^ plasma cells in the spleen of YM-58483- or vehicle-treated mice. **(C)** Anti-dsDNA antibodies (anti-dsDNA) in the sera of YM-58483- or vehicle-treated mice was measured by ELISA and shown as dot plot with bar. **(D)** Kidney samples from YM-58483- or vehicle-treated mice were examined with a Tecnai G2 Spirt Twin electron microscope. Representative images are shown, and immune complex deposition in subendothelial area of glomeruli is indicated by red arrows. Scale bar 1μM. **(E, F)** OCT-embedded kidney samples from YM-58483- or vehicle-treated mice were sectioned and stained with antibodies against IgG and C3. Sections were counterstained with DAPI and visualized using a fluorescence microscopy. Representative images are shown. Fluorescence intensity of IgG or C3 is summarized and shown as dot plot with bar plot. n = 7 in vehicle group and n = 8 in YM-58483 group. Scale bar 50μM. All data are mean ± SEM. *p < 0.05, **p < 0.01, and ***p < 0.001 by t-test.

To further investigate the role of CRAC channel in B-cell differentiation and LN development *in vivo*, MRL/*lpr* mice were treated with YM-58483 from 12 weeks of age after the development of proteinuria as described in *Methods and Materials*. Percentage of B220^−^CD138^+^ plasma cells in the spleens of YM-58483-treated mice were significantly lower than those treated with vehicle as determined by flow cytometry (**Figures 5A, B**). Tfh cells play a critical role in GC reaction and B-cell differentiation into plasm cells. However, the percentages of Tfh cells and GC B cells were not affected by YM-58483 (**Supplementary Figures S6A–D**). The percentages of CD4^+^, CD8^+^, B220^+^, and CD11b^+^ cells in the spleens were not different between YM-58483 or vehicle-treated groups neither (**Supplementary Figures S6E–L**), indicating that the homeostasis of these immune cells in the spleen was not affected by YM-58483 treatment.

Anti-dsDNA are produced by autoreactive B cells or plasma cells. We found that the concentration of anti-dsDNA in the sera was decreased from 25.3 to 12.4 ng/ml by YM-58483 (**Figure 5C**). Additionally, immune complex deposition in subendothelial area of glomeruli was reduced by YM-58483 as determined by transmission electron microscopy (TEM) (**Figure 5D**). IgG and C3 deposition in the glomeruli were decreased profoundly by YM-58483 as measured by immunofluorescence (**Figures 5E, F**). To further investigate whether CRAC channel inhibition can prevent the immune complex deposition to the kidney, MRL/*lpr* mice were treated with YM-58483 from 8 weeks of age before the development of proteinuria. We found that the deposition of immune complex to the kidney was greatly prevented by YM-58483 (**Supplementary Figure S7**).

### YM-58483 Ameliorates Kidney Damages and Improved Renal Function in MRL/*lpr* Mice

**Figure 6:**
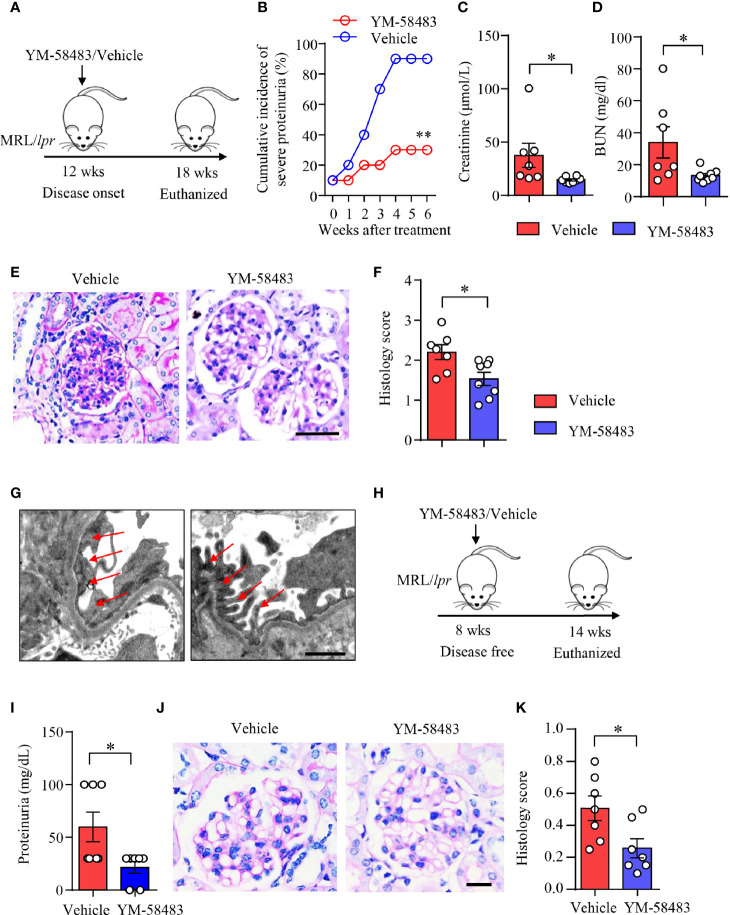
YM-58483 ameliorated kidney damage and improved renal function in MRL/lpr lupus mice. **(A)** MRL/lpr mice (12-week) were treated with YM-58483 (1 mg/kg) or vehicle intraperitoneal once a day for 6 weeks. **(B)** Severity of proteinuria of YM-58483- or vehicle-treated mice were monitored and recorded weekly. **(C, D)** Concentrations of creatinine or blood urea nitrogen (BUN) in the sera. **(E)** Kidney samples from YM-58483- or vehicle-treated mice were stained with PAS. Scale bar 50μM. **(F)** Histology score for PAS staining was summarized, n = 7 in the vehicle group and n = 8 in the YM-58483 group. **(G)** Kidney samples were prepared for transmission electron microscopy to assess the pathological features of glomeruli. Representative images are shown. Red arrows indicate foot process of podocytes. Scale bar 1μM. **(H)** MRL/lpr mice (8-week) were treated with YM-58483 (1 mg/kg) or vehicle intraperitoneal once a day for 6 weeks. **(I)** Proteinuria of YM-58483- or vehicle-treated mice were recorded in 14 weeks. **(J)** Kidney samples from YM-58483- or vehicle-treated mice were stained with PAS to assess the pathological features of glomeruli. Scale bar 20μM. **(K)** Histology score for PAS staining was summarized. n =7 in the vehicle group and n = 7 in the YM-58483 group. All data are mean ± SEM. *p < 0.05, **p < 0.01 by t-test.

MRL/*lpr* mice were treated with YM-58483 when disease was established as described (**Figure 6A**). We found that the severity of proteinuria was significantly reduced by YM-58483. By the end of treatment, 90% of the mice from vehicle group developed severe proteinuria, and only 30% of the mice from YM-58483 group developed severe proteinuria (**Figure 6B**). Renal function was improved in YM-58483-treated mice. The levels of creatinine and BUN in the sera were decreased by YM-58483 significantly (**Figures 6C, D** **)**. YM-58483 treatment attenuated cellular proliferation and infiltration profoundly as assessed by PAS staining (**Figures 6E, F** **)**. TEM imaging also showed decreased effacement of podocyte foot process by YM-58483 treatment (**Figure 6G**). To further investigate whether CRAC channel play a role during disease initiation, MRL/*lpr* mice were treated with YM-58483 at the age of 8 weeks before the development of proteinuria (**Figure 6H**). We found that YM-58483 treatment prevented the development of proteinuria (**Figure 6I**) and nephritis in MRL/*lpr* mice (**Figures 6J, K**).

### YM-58483 Decreases Immune Deposition and Ameliorates Kidney Damages in Lupus Mice of NZM2328

**Figure 7:**
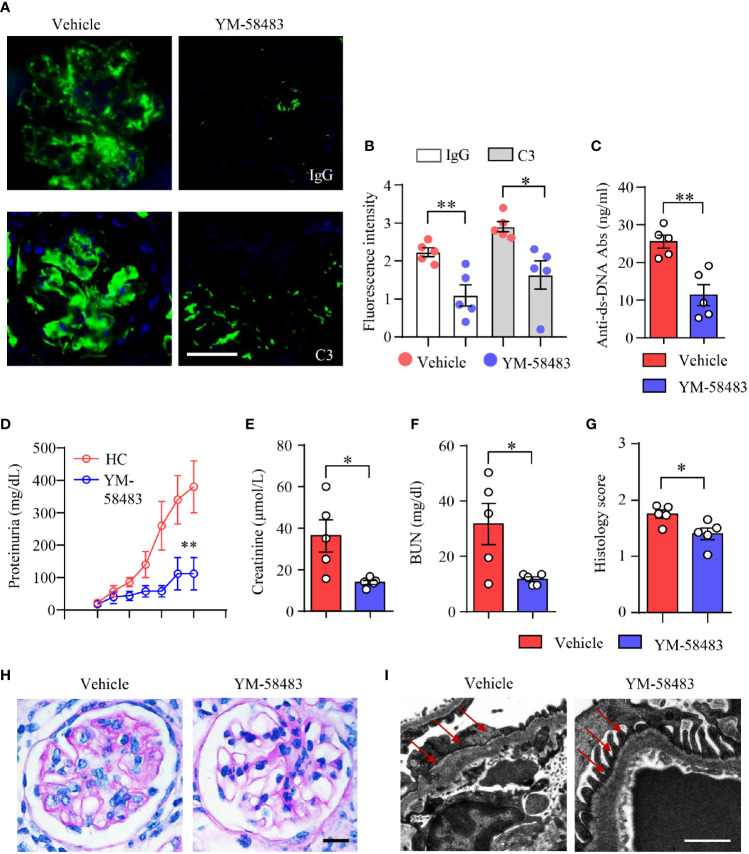
YM-58483 reduced immune deposition and ameliorated kidney damage in NZM2328 lupus mice. NZM2328 mice were treated with YM-58483 (1 mg/kg) or vehicle intraperitoneal once a day for 6 weeks. **(A, B)** OCT-embedded kidney samples from YM-58483- or vehicle-treated mice were sectioned and stained with antibodies against IgG and C3. Sections were counterstained with DAPI and visualized using a fluorescence microscopy. Representative images are shown. Fluorescence intensity of IgG or C3 were summarized and shown as dot plot with bar plot. Scale bar 50μM. **(C)** Anti-dsDNA antibodies (anti-dsDNA) in the sera of YM-58483- or vehicle-treated mice was measured by ELISA and shown as dot plot with bar. **(D)** Proteinuria of YM-58483- or vehicle-treated mice were monitored and recorded weekly. **(E, F)** Concentrations of creatinine or blood urea nitrogen (BUN) in the sera. **(G, H)** Kidney samples from YM-58483- or vehicle-treated mice were stained with PAS. Histology score for PAS staining was summarized. Representative images were shown. Scale bar 20μM. **(I)** Kidney samples were prepared for transmission electron microscopy to assess the pathological features of glomeruli. Electron microscope images. Red arrows indicate foot process of podocytes. Scale bar 0.5μM. n = 5 in the vehicle group and n = 5 in the YM-58483 group. All data are mean ± SEM. *p < 0.05, **p < 0.01 by t-test.

To further confirm the therapeutic effects of CRAC channel inhibition over LN, another lupus mouse model NZM2328 was adopted and treated with CRAC channel inhibitor YM-58483. Immune deposition in the glomeruli was first evaluated by immunofluorescence. In consistent with data from MRL/*lpr* mice, YM-58483 greatly reduced immune complex deposition in the glomeruli. IgG and C3 were found to be significantly less in the glomeruli of NMZ2328 mice treated by YM-58483 when compared to the vehicle group (**Figures 7A, B**). The autoantibody of anti-dsDNA was measured by ELISA, the result revealed that the concentration of anti-dsDNA in the sera of NZM2328 mice was reduced by YM-58483 significantly (**Figure 7C**). Next, we were to evaluate renal function and pathological changes in YM-58483 or vehicle-treated mice. We found that YM-58483 prevented the progression of proteinuria in NZM2328 mice effectively (**Figure 7D**). The levels of creatinine and BUN in the sera of mice were lower in YM-58483-treated mice (**Figures 7E, F**). Furthermore, YM-58483 treatment decreased cellular proliferation of glomeruli as assessed by PAS staining (**Figures 7G, H**). TEM was applied for kidney damage evaluation, and the result showed decreased effacement of podocyte foot process in YM-58483-treated NZM2328 mice (**Figure 7I**), which further confirmed the therapeutic effects of YM-58483 over the progression of LN.

## Discussion

The development of novel therapies for lupus nephritis (LN) has been hampered by the poor understanding of disease pathogenesis. Progress has been made in the past two decades in the field of B-cell targeted therapy in SLE (42).

By searching the public databases, RNA-seq analysis, using patient blood samples and mouse models, we found that the expression of CRAC channel and Ca^2+^ influx was increased in B cells from patients with LN. Downstream calcium signals as mediated by CaM/CaMK2 were also enhanced. CRAC-channel-mediated SOCE is the major mechanism that controls Ca^2+^ influx in lymphocytes (43). BCR stimulation leads to increased IP3 production, which mediates Ca^2+^ release to trigger SOCE in B cells (9, 44). A large variety of signaling cascades are known to participate in the generation and modulation of calcium signals in B cells as mediated by BCR stimulation. BCR-triggered calcium signals are essential for B-cell development and activation (45). A gain-of-function mutation has been identified in the murine *Plcg2* gene, which leads to hyperreactive Ca^2+^ entry in B cells and results in severe spontaneous autoimmune inflammation (11). Mutations in the *BLNK* gene, an essential component for the generation of calcium signaling by BCR, resulted in humoral immunodeficiency for the defect of early B-cell development in the bone marrow (46). The increased CRAC channels in B cells could contribute to B-cell dysregulation and cause kidney damage in LN.

In the current study, we found that store-operated CRAC channel and the associated calcium signaling were particularly enhanced in naive B cells from patients with LN when compared to other B-cell subsets. We also detected decreased naive B cells in patients with LN. In considering the important roles of DN B cells in SLE (47), we also found an expansion of DN B cells in patients with LN. However, CRAC channel in DN B cells was not different compared to that from HC. These data demonstrate that CRAC channel promotes early activation of B cells in patients with LN, pinpointing an important role of CRAC channel in the initiation and development of LN. It has been shown that patients with mutations in genes of *ORAI1* and *STIM1* show little SOCE but normal B-cell numbers (48). It seems that SOCE is dispensable for B-cell development. However, other immunes, like T cells, NK cells, and monocytes, are also defective in these patients. It is difficult to speculate the specific functions of SOCE over B cells in these patients with mutations.

Blimp-1 is the key transcription factor for the differentiation of plasma cells (40, 49). We found that Blimp-1 was upregulated in B cells from LN patients. Naive B cells from patients with LN were prone to differentiate into plasma cells. CRAC channel inhibition or knockdown of ORAI1 suppressed Blimp-1 expression and decreased the differentiation of IgG-producing plasma cells, which is in consistent with the previous study that CRAC inhibitors attenuate the function of B cells from patients with RA (50). However, CRAC channel inhibition did not affect proliferation of human B cells. This might be due to the stimulation of CD40 on B cells in our cell culture system, which was in accordance with the previous study that CD40 stimulation can bypass the inhibition over CRAC channel and rescue B cell proliferation (51).

Calcium functions as a universal second messenger in basically all eukaryotic cells, including B cells, T cells, and other immune cells. BCR stimulation leads to the activation of SOCE and influx of Ca^2+^ (52). Ca^2+^ influxes through CRAC channels and activates Ca^2+^-dependent enzymes, such as calcineurin and CaM/CaMK, and thereafter transcription factors, such as nuclear factor of activated T cell (NFAT) and nuclear factor kappa B (NF-κB) (53, 54). RNA-seq data in this study revealed that NFAT, NF-κB, and TOR signaling pathways were not affected by YM-58483, pointing to the dispensable role of CRAC channels in controlling these pathways in human B cells (**Supplementary Figure S8**). Interestingly, calcium signaling pathway mediated by CaM/CaMK2 was enriched by YM-58483-treated B cells (**Supplementary Figure S8**). The Western blot analysis showed that CRAC channel inhibition suppressed the phosphorylation of CaMK2. The enriched gene set of calcium signaling pathway at the RNA level should be the result of a compensatory reaction following the inhibition of CaMK2. Combined together, these data demonstrated that CRAC channel controlled calcium signaling pathway through CaM/CaMK2 in human B cells. CaMK2 could play an important role in the function and differentiation of human B cells.

The roles of CaMK2 in LN is not clear. In this study, we found that phosphorylation of CaMK2 was upregulated in B cells from LN patients. CaMK2 inhibitor or knockdown of CaMK2 downregulated Blimp-1 expression in B cells and led to the decreased differentiation of IgG-producing plasma cells. These data suggest that CaMK2 could be involved with the pathogenesis of LN by controlling B cell activation.

Data in this study showed that CRAC channel was increased in B cells from patients with LN and that CRAC channel might promote the development of LN by controlling B cell differentiating into IgG-producing plasma cells. Lupus mice treated with CRAC channel inhibitor YM-58483 were found to have less plasma cells in the spleens. The concentration of anti-dsDNA was significantly decreased in YM-58483-treated mice. The inhibition of plasma cell differentiation and decreased anti-dsDNA production were in consistent with reduced immune deposition in the glomeruli of lupus mice, which led to improved renal function as by CRAC channel inhibition. The percentage of other immune cell linages including CD4^+^ T cells, CD8^+^ T cells, B220^+^ B cells, and CD11b^+^ monocytes were largely unaffected. Previous study showed that conditional knockout of *STIM1*/*STIM2* in T cells led to impaired antibody response for the defect of Tfh development (55). CRAC channel inhibition also decreased antibody production in a humanized chimeric mouse model for RA in another study (50). The difference might be due to the disease model used and the relatively low dosage of the YM-58483 inhibitor (56) in the current study. These data demonstrate that CRAC channel controls B-cell differentiation independent of help from Tfh cells in lupus mice.

It has been revealed that conditional knockout of *STIM1*/*STIM2* in B cells showed no effect on B-cell development (51). However, the authors did not analyze the effects of CRAC channel on the development of GC B cells into memory B cells or plasma cells in that study. It was not clear whether CRAC channel affected B-cell differentiation in that study or not. In the current study, B220^+^ B cells and GC B cells were not affected by CRAC channel inhibition neither. We speculate that store-operated CRAC channel might mediate the differentiation of B cells into plasma cells but does not affect B-cell development and B-cell differentiation before entering GC. The true roles of CRAC channel in B cells could also be different under disease conditions. What we need to point out in this study is that we did not see the difference in total CD4^+^/CD8^+^ T cells in the spleens of YM-58483-treated mice. We did not analyze the subsets of these T cells. YM-58483 could act on some specific T cell subsets with an indirect effect on B cells.

In conclusion, our results suggested that store-operated CRAC channel controls the differentiation of B cells into plasma cells through CaMK2. CRAC channel inhibition is effective in protecting against LN by inhibiting the differentiation of pathogenic B cells. CRAC channel could serve as a potential therapeutic target for LN.

## Data Availability Statement

The datasets presented in this study can be found in online repositories. The names of the repository/repositories and accession number(s) can be found in the article/**Supplementary Material**.

## Ethics Statement

The studies involving human participants were reviewed and approved by Institutional Ethical Committee of First Affiliated Hospital, Sun Yat-sen University. The patients/participants provided their written informed consent to participate in this study. The animal study was reviewed and approved by Ethics Committee of First Affiliated Hospital of Sun Yat-sen University.

## Author Contributions

XL, QZ, SW, HZ, and NY conceived and designed the study. XL, QZ, SW, ML, and XC recruited patients and collected and analyzed clinical data. XL, QZ, SW, ML, XC, YH, BC, MZ, YL, CG, and SZ performed the experiments. XL, QZ, SW, ML, XC, YH, BC, MZ, YL, CG, SZ, HZ, and NY analyzed and interpreted the experiment data. XL, HZ, and NY drafted the manuscript, and all authors participated in writing the final manuscript. HZ and NY approved and supervised the project. All authors contributed to the article and approved the submitted version.

## Funding

This work is supported by the National Natural Science Foundation of China (81971519, 81671593, 81471598, 81701595, and 82071819), Guangzhou Science and Technology Planning Program (201707010093), and National Key Research and Development Project (2017YFC0907602).

## Conflict of Interest

The authors declare that the research was conducted in the absence of any commercial or financial relationships that could be construed as a potential conflict of interest.

## Publisher’s Note

All claims expressed in this article are solely those of the authors and do not necessarily represent those of their affiliated organizations, or those of the publisher, the editors and the reviewers. Any product that may be evaluated in this article, or claim that may be made by its manufacturer, is not guaranteed or endorsed by the publisher.
