# Supplementary material for: CRAC Channel Controls the Differentiation of Pathogenic B Cells in Lupus Nephritis
Source: Front Immunol. 2021 Oct 22;12:779560. doi: 10.3389/fimmu.2021.779560 (PMC8569388; doi:10.3389/fimmu.2021.779560)
Supplement: Supplementary file 1 [file DataSheet_1.pdf]

Supplementary Material

Supplementary Table 1. **Demographics of the LN patients in this study**

Parameters	Patients (n=60)	References
Age, year (mean ±SEM)	29.3 ±1.4	
Female/male (no.)	48/12	
Newly-onset (no.）	28/32	
Time since onset, year	1.4 ±0.1	
SLEDAI	12.3 ±0.9	
ESR (mm/h)	44.3 ±3.6	(≤34)
ANA（U/ml）	186.40 ±14.4	(0-12.00)
Anti-ds-DNA antibody (IU/ml)	151.4 ±15.97	(0-30.00)
C3 (g/L)	0.52 ±0.03	(0.79-1.17)
C4 (g/L)	0.11 ±0.01	(0.17-0.31)
IgG (g/L)	14.0 ±0.98	(10.13-15.13)
IgA (g/L)	1.79 ±0.11	(1.45-3.45)
IgM (g/L)	0.86 ±0.08	(0.92-2.04)
ALB (g/L)）	29.38 ±0.97	(35-50)
Creatinine (μmol/L)	87.66 ±9.14	(53-115)
Proteinuria (g/24h)	3.44 ±0.56	(0-0.12)

Supplementary Figures and legends

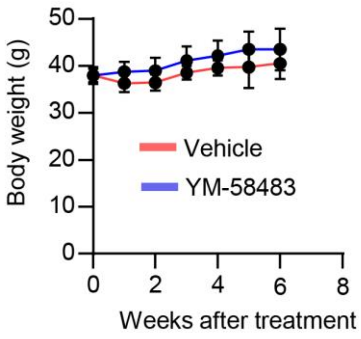

Supplementary Fig. 1. **Body weight kinetics of YM-58483 or vehicle treated MRL/*lpr* mice**.

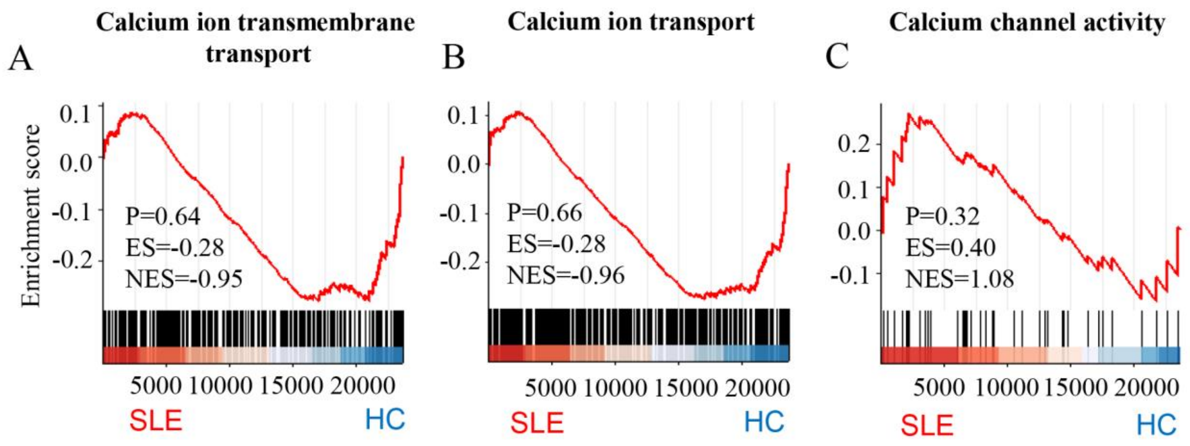

Supplementary Fig. 2**. Enhanced calcium transmembrane transport and calcium signaling pathway in B cells from LN patients.**) RNA-seq data of CD4 T cells of systemic lupus erythematosus (SLE) or healthy controls (HC) were acquired from data bases (GSE120442) for gene expression and bioinformatics analysis. (A-C) Gene Set Enrichment Analysis (GSEA) plots of (A) calcium transmembrane transport (“GOMP_CALCIUM_ION_TRANSMEMBRANE_TRANSPORT”), (B) calcium ion transport (“GOMP_CALCIUM_ION_TRANSPORT”) and calcium channel activity ("GOMF_CALCIUM_CHANNEL_REGULATORY_ACTIVITY"). Enrichment scores (ES) are shown on the y-axis. Positive and negative ES indicate enrichment in SLE and HC samples, respectively. X-axis (black vertical lines) represent individual genes of the gene set. The colored band on the bottom represents the degree of correlation of genes with SLE phenotype (red for positive correlation and blue for negative).

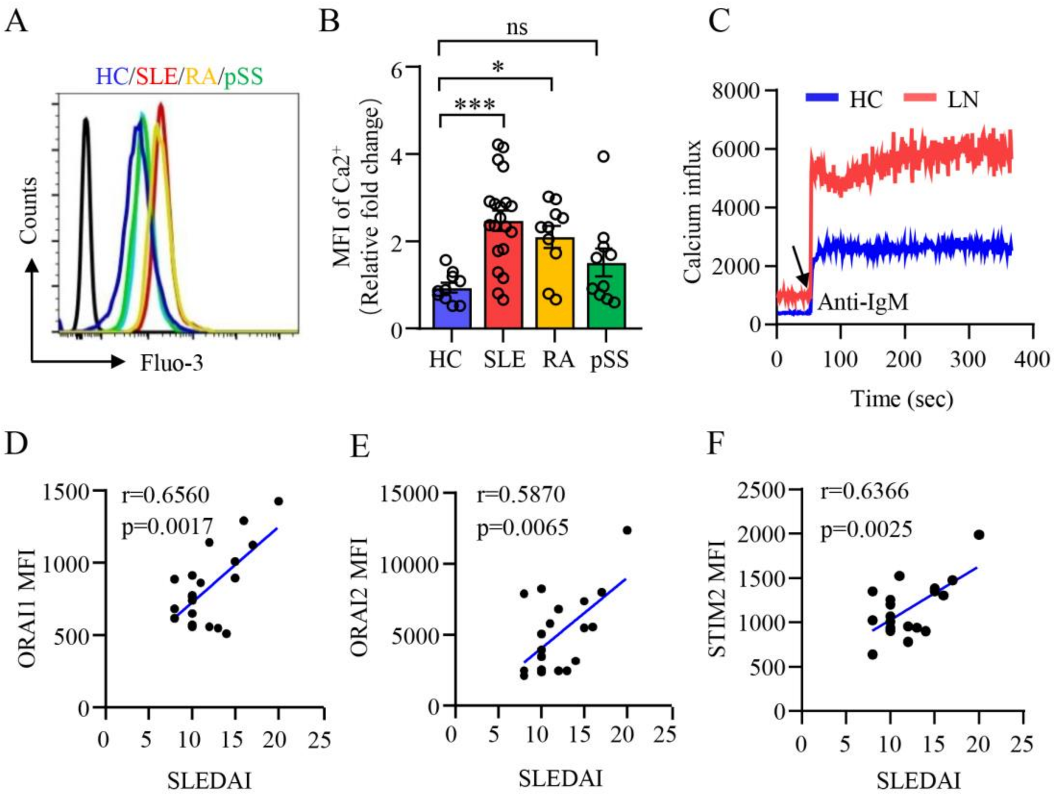

Supplementary Fig. 3. **Enhanced calcium level in B cells from LN patients and the correlation of CRAC channel with disease activities**. (A) PBMC isolated from patients with lupus nephritis (LN), rheumatoid arthritis (RA) and primary Sjögren‘s syndrome (pSS) were stained with antibodies against CD19, CD27 and IgD. Cells were then labeled with Fluo-3 and measured by flow cytometry. Representative histograms were gated on $CD19^+$ B cells. (B) Mean fluorescence intensity (MFI) of Fluo-3 in $CD19^+$ B cells from LN patients (n=20), RA (n=10), pSS (n=10) or HC (n=10) were summarized and shown as dot plot with bar. (C) B cells were isolated from LN patients or HC and labeled with calcium indicator Fluo-3 AM. Calcium influx was monitored by flow cytometry as stimulated by anti-IgM antibody. Representative calcium influx kinetics of 3 samples were shown. (D-F) SLEDAI was calculated for each LN patients and the correlations between the expression of ORAI1, ORAI2 and STIM2 and SLEDAI were shown (n=20). Data are mean ± SEM. *$p<0.05$, ***$p<0.001$ by one-way ANOVA. ns: not significant.

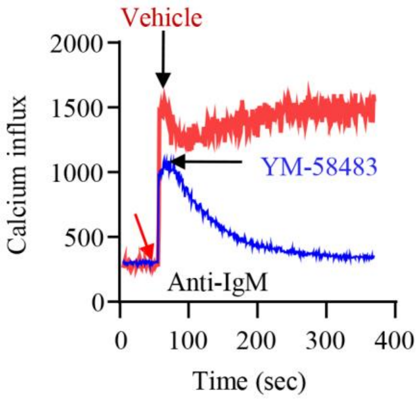

Supplementary Fig. 4. **YM-58483 inhibited Ca^{2+} influx into B cells**. B cells isolated from healthy controls were labeled with Ca^{2+} indicator Fluo-3 AM. Ca^{2+} influx was monitored by flow cytometry as stimulated by anti-IgM antibody. YM-58483 (1μM) or vehicle was added during the recording of calcium influx (red arrow). Representative Ca^{2+} influx kinetics of 3 independent samples.

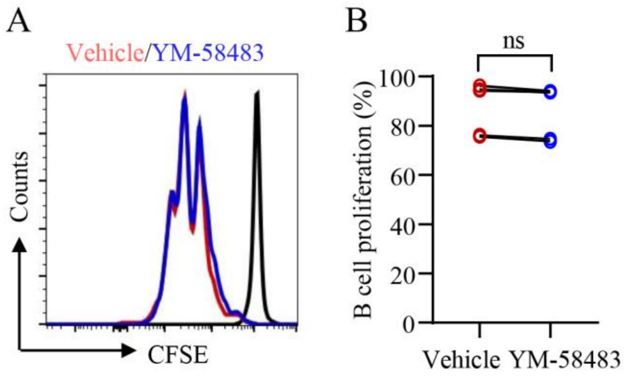

Supplementary Fig. 5. **YM-58483 and KN-93 showed no effects on B cell proliferation**. B cells isolated from PBMC of healthy controls were labeled with CFSE. Cells were then stimulated with anti-IgM (5µg/ml), anti-CD40 (1µg/ml) in the presence of YM-58483 (50nM) or KN-93(5uM) or vehicle for 4 days. Cells were then collected and B cell proliferation was measured by flow cytometry. (A-D) Representative histograms are shown and data summarized from 5 independent samples. All data are mean ± SEM. ns: not significant by paired t test.

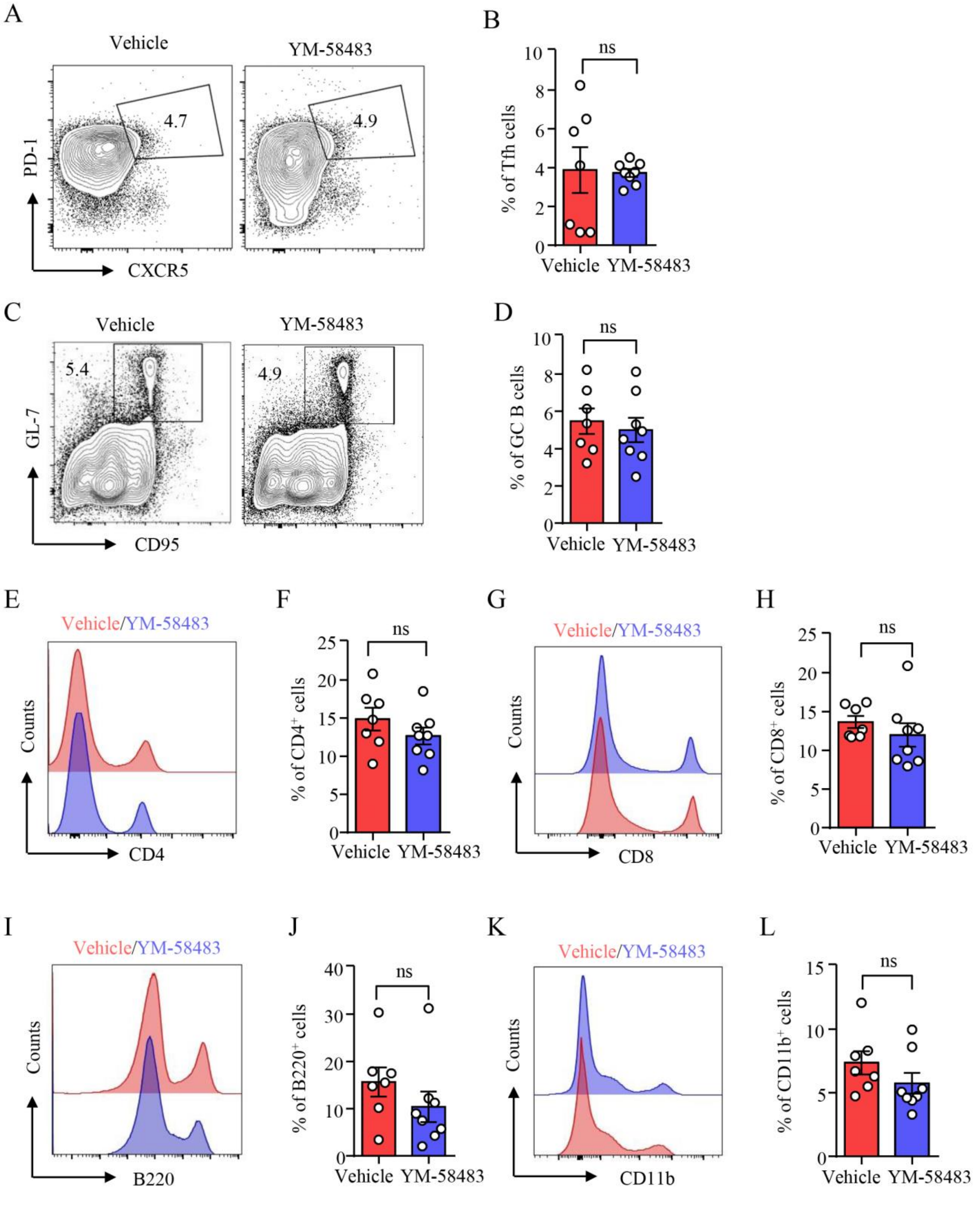

Supplementary Fig. 6. **YM-58483 did not affect homeostasis of immune cells in the spleen of MRL/*lpr* lupus mice**. MRL/*lpr* mice were treated with YM-58483 (1mg/kg) or vehicle intraperitoneally once a day for 6 weeks. Spleens were collected and single cells were prepared when

the treatment was finished. (A-L) Cells were stained with antibodies against CD4, CD8, B220, CD11b, PD-1, CXCR5, GL-7 and CD95. Samples were measured by flow cytometry. Representative FACS plots of follicular helper T cells (Tfh): $CXCR5^{+}PD\text{-}1^{+}$, germinal center (GC) B cells: $GL\text{-}7^{+}CD95^{+}$, $CD4^{+}$ T cells, $CD8^{+}$ T cells, $B220^{+}$ B cells and $CD11b^{+}$ monocytes are shown. n=7 in vehicle group and n=8 in YM-58483 group. All data are mean ± SEM. ns: not significant by t test.

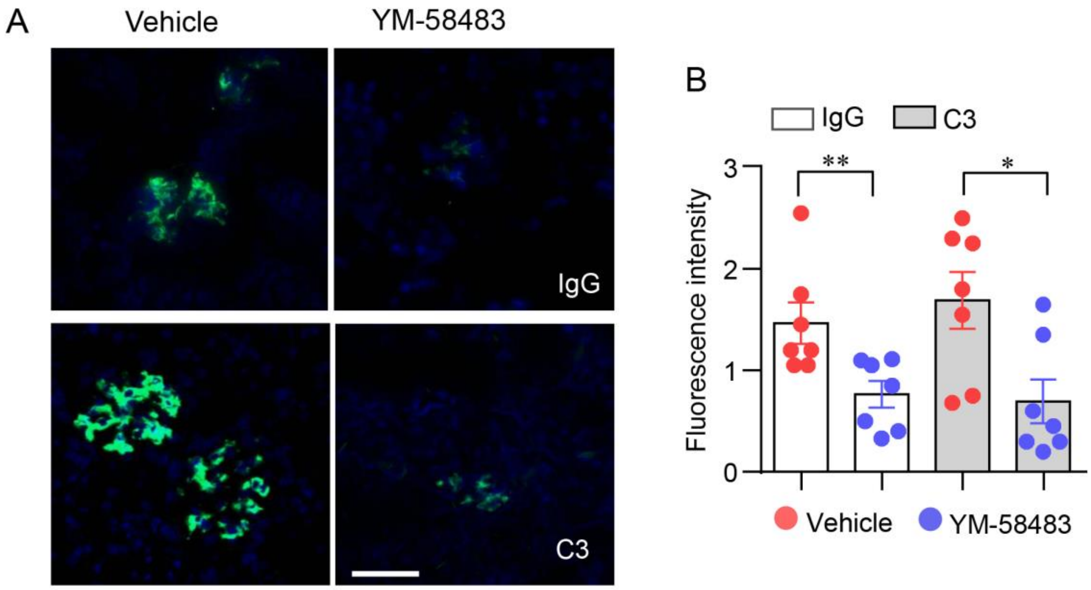

Supplementary Fig. 7. **YM-58483 prevented immune deposition in the kidney of MRL/*lpr* lupus mice**. MRL/*lpr* lupus mice (8 weeks old) were treated with YM-58483 (1mg/kg) or vehicle intraperitoneal once a day for 6 weeks. (A, B) O.C.T embedded kidney samples from YM-58483 or vehicle treated mice were sectioned and stained with antibodies against IgG and C3. Sections were counter stained with DAPI and visualized using a fluorescence microscopy. Representative images were shown. Fluorescence intensity of IgG or C3 were summarized and shown as dot plot with bar plot. Images are 400× magnification. Scale bar: 25μm. All data are mean ± SEM. *p<0.05 and ** p<0.01 by t test.

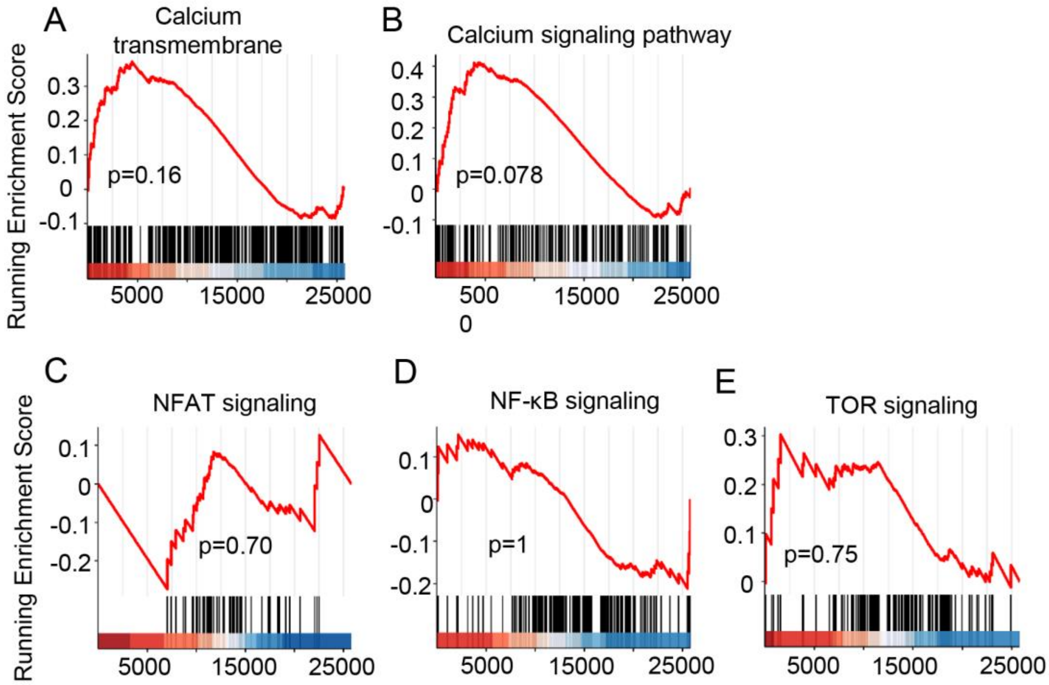

Supplementary Fig. 8. **Gene Set Enrichment Analysis (GSEA) plots of:** (A) calcium transmembrane transport (“GO_CALCIUM_ION_TRANSMEMBRANE_TRANSPORT”); (B) calcium signaling pathway (“KEGG_CALCIUM_SIGNALING_PATHWAY”); (C) NFAT signaling pathway (“PID_NFAT_3PATHWAY”); (D) NIK/NF-kappaB signaling pathway (“GO_NIK_NF_KAPPAB_SIGNALING”); (E) TOR signaling pathway (“GO_TOR_SIGNALING”). Enrichment scores (ES) are shown on the y-axis. Positive and negative ES indicate enrichment in YM-58483 and vehicle samples, respectively. X-axis (black vertical lines) represent individual genes of the gene set. The colored bands on the bottom represent the degree of correlation of genes with YM-58483 phenotype (red for positive correlation and blue for negative).
